# Short- and Long-Term Advantages of Laparoscopic Gastrectomy for Elderly Patients with Locally Advanced Cancer

**DOI:** 10.3390/cancers16132477

**Published:** 2024-07-07

**Authors:** Francesco Puccetti, Lorenzo Cinelli, Stefano Turi, Davide Socci, Riccardo Rosati, Ugo Elmore, on behalf of the OSR CCeR Collaborative Group

**Affiliations:** 1 Department of Gastrointestinal Surgery, IRCCS San Raffaele Scientific Institute, 20132 Milan, Italy; cinelli.lorenzo@hsr.it (L.C.); socci.davide@hsr.it (D.S.); rosati.riccardo@hsr.it (R.R.); elmore.ugo@hsr.it (U.E.); 2 School of Medicine, Vita-Salute San Raffaele University, 20132 Milan, Italy; 3 Department of Anesthesia and Intensive Care, IRCCS San Raffaele Scientific Institute, 20132 Milan, Italy; turi.stefano@hsr.it

**Keywords:** gastrectomy, gastric cancer, frailty, laparoscopy, postoperative complications

## Abstract

**Simple Summary:**

The aging population has led to an increase in elderly patients with locally advanced gastric cancer (LAGC) requiring in-hospital care. These patients often present with higher levels of comorbidity and frailty, due to reduced physiological reserve and functional capacity, with a major susceptibility to surgery-related complications. However, the adoption of specific ERAS guidelines on gastric cancer surgery for elderly patients has been proven safe and effective, leading to optimize hospital stay and costs with equal complication rates. Prehabilitation programs also improve pre-treatment patient function, potentially enhancing their capacity to tolerate multimodal therapy. This study explores whether elderly patients affected by LAGC following an ERAS-based protocol may benefit from laparoscopic procedures, which could reduce operative stress and possibly improve survival in frail individuals.

**Abstract:**

Minimally invasive surgery has provided several clinical advantages in locally advanced gastric cancer (LAGC) care, although a consensus on its application criteria remains unclear. Surgery remains a careful choice in elderly patients, who frequently present with frailty, comorbidities, and other disabling diseases. This study aims to assess the possible advantages of laparoscopic gastric resections in elderly patients presenting with LAGC. This retrospective study analyzed a single-center series of elderly patients (≥75 years) undergoing curative resections for LAGC between 2015 and 2020. A comparative analysis of open versus laparoscopic approaches was conducted, focusing on postoperative complications, length of hospital stay (LOS), and long-term survival. A total of 62 patients underwent gastrectomy through an open or a laparoscopic approach (31 pts each). The study population did not show statistically significant differences in demographics, operative risk, and neoadjuvant chemotherapy. The laparoscopic group reported significantly minimized overall complications (45.2 vs. 71%, *p* = 0.039) and pulmonary complications (0 vs. 9.7%, *p* = 0.038) as well as a shorter LOS (8 vs. 12 days, *p* = 0.007). Lymph node harvest was equal between the groups, although long-term overall survival presented significantly better after laparoscopic gastrectomy (*p* = 0.048), without a relevant difference in terms of disease-free and disease-specific survivals. Laparoscopic gastrectomy proves effective in elderly LAGC patients, offering substantial short- and long-term postoperative benefits.

## 1. Introduction

Despite its steady decline in incidence, gastric cancer (GC) remains the fifth most prevalent malignancy and carries the fourth highest cancer-related mortality rate worldwide, with an estimated 5-year overall survival (OS) lower than 20–30% [1,2,3]. Although GC epidemiology is highly variable by dietary risk and geographical factors, such as the regional prevalence of Helicobacter pylori infection, a cumulative risk has been reported with increasing age, starting from 45 years and reaching a median age of diagnosis of 69–70 years [2,4]. In addition to the relationship between cancer development and advancing age, the global aging of patients requiring in-hospital care has led to further increased proportions of elderly people presenting with locally advanced gastric cancer (LAGC) [5]. This particular class of patients is reportedly more likely to present with higher comorbidity levels and frailty, which creates an age-related multidimensional condition characterized by reduced physiological reserve and multifunctional capacity. Therefore, frailty has been associated with higher individual susceptibility to the augmented occurrence and severity of complications after surgery, including GC resections [6,7,8,9]. The poor outcomes of gastric surgery in the elderly have historically limited indications of invasive treatments to frail patients, while such an increasing public health issue may, conversely, present acceptable results if addressed with dedicated resources and new settings of operative stress minimization.

Some of the recently validated strategies for operative stress minimization include the standardized perioperative protocols of Enhanced Recovery After Surgery (ERAS) and minimally invasive surgery. After the development of the ERAS consensus guidelines for gastrectomy in 2014 [10], the transition to elderly patients of this multidisciplinary and function-based management has been largely demonstrated to be safe and effective in optimizing the length of hospital stay (LOS) and costs without increasing postoperative complications [11,12]. Prehabilitation programs have also allowed patients to improve their functional status before treatments, potentially facilitating their access and tolerance to the different components of multimodal therapy for LAGC.

On the other hand, further short- and long-term benefits have been reported following the implementation of minimally invasive techniques in GC surgery [13]. After its early diffusion in the late ‘90s [14], laparoscopic gastrectomy (LG) for cancer treatment initially spread among high-volume centers for the treatment of early-stage disease and distal tumors [15,16]. Despite the large debate concerning the laparoscopic accuracy for LAGC resections, RCTs’ scientific evidence (i.e., CLASS-01, KLASS-02, LOGICA, STOMACH trials) has recently been supporting the safety and efficacy of laparoscopy for the treatment of GC that is of a locally advanced stage or has been previously treated with chemotherapy. According to the literature, postoperative morbidity, lymph node harvest, and OS are demonstrated to be summarily comparable between laparoscopic and conventional surgery [17,18,19,20].

The present study hypothesized that elderly patients undergoing LAGC resections within an ERAS-based standardized protocol might benefit from the application of laparoscopy and that the minimization of operative stress due to laparoscopy may also impact survival of frail patients. Therefore, this analysis aimed to compare both short-term outcomes and 2- and 5-year survival rates of elderly patients undergoing either laparoscopic or open LAGC surgery.

## 2. Materials and Methods

The present study was conducted in accordance with the STrengthening the Reporting of OBservational studies in Epidemiology statement (STROBE) guidelines (Table S1) [21].

### 2.1. Study Design

This analysis included a single-center series of consecutive patients who underwent gastrectomy with D2 lymphadenectomy between January 2015 and December 2020 at a tertiary center (San Raffaele Research Hospital—Milan, Italy).

All cases were retrospectively selected from an IRB-approved prospectively maintained institutional database, according to the following inclusion criteria: histologically confirmed diagnosis of locally advanced gastric adenocarcinoma (T ≥ 2, any N, M0); either total or distal gastrectomy with curative intent, according to the European Society for Medical Oncology (ESMO) clinical practice guidelines [22]; either laparoscopic or open surgical approach; aged ≥ 75 years; either neoadjuvant therapy or upfront surgery; and standardized management including perioperative multidisciplinary assessments and ERAS-based clinical protocol, with overall compliance as high as 80% at least [10]. Locally advanced gastric cancers were preoperatively staged through total-body CT-scan and a diagnostic laparoscopy (DL) with peritoneal lavage for microscopic metastases. Also, the multidisciplinary team assessment (Figure S1) was routinely scheduled to evaluate preoperative findings and provide indications for additional investigations.

Exclusion criteria were any histological subtypes other than adenocarcinoma, any resection types other than total or distal gastrectomy, and multivisceral resections. Histological confirmation was generally provided with microbiological findings of Helicobacter Pylori according to the endoscopic depiction, although these data were not constant.

Laparoscopy represented the surgical approach of choice unless preoperative findings were consistent with technical limitations (i.e., bulky dimensions of the primary tumor or suspicion of infiltration of surrounding organs) or patient-related impediments for laparoscopic surgery (i.e., history of previous surgery or intolerance to 12 mmHg pneumoperitoneum). Patients who required conversion from laparoscopic to open approach were included in the laparoscopic group based on the intention-to-treat principle. All procedures were performed by two of authors (RR and UE) who shared the technique standardization, including the equivalent extent of lymphadenectomy and anastomosis fashioning [23]. All patients were postoperatively managed according to a standardized ERAS-based perioperative program (Figure S1). The primary outcomes were postoperative complications, LOS, and long-term OS. The impact of laparoscopy on mean operative time, lymph node harvest, and 30-day readmission rate was secondarily assessed.

### 2.2. Surgery, Perioperative Management, and Follow-Up

Patients were submitted to either total or distal gastrectomy according to tumor location and the degree of cell differentiation [22]. Gastrectomy was performed through either laparoscopic or open surgery, depending on both disease and patient characteristics. Laparoscopy was implemented through induction of pneumoperitoneum at 12 mmHg and a four-port technique with a mini-suprapubic incision. Open surgery involved an upper midline laparotomy, and the patient was supine in both cases. In total gastrectomy, a side-to-side (linear stapler) and end-to-side (circular stapler) esophago-jejunal anastomoses were fashioned through laparoscopic and open approaches, respectively. In distal gastrectomy, both techniques routinely involved a Roux-en-Y reconstruction with a jejuno-jejunal anastomosis performed 50–60 cm below the former, which was side-to-side (mechanical) in laparoscopic resections, or end-to-side (hand-sewn) in open surgery. Also, the duodenum was routinely stapled without further oversewn stitches to avoid altering the stump perfusion.

The standardized ERAS-based perioperative protocol was uniformly applied throughout the study period, including all main perioperative items potentially enhancing recovery (Table S2). The choice of complementary therapies, indications for surgery, and type of follow-up monitoring were undertaken by a multidisciplinary board.

During the first 12 months after surgery, routine blood tests, upper GI endoscopy, and chest/abdomen computed tomography (CT) were performed periodically (i.e., in 6-month periods after resection and every 12 months afterward).

### 2.3. Data Definition and Collection

All clinical data were retrospectively collected from an institutional, IRB-approved, and prospectively maintained electronic database. Perioperative data included patients’ demography (i.e., age, gender, body mass index [BMI], age-adjusted Charlson comorbidity index [aa-CCI] [24], the American Society of Anesthesiologists [ASA] score [25], and the Nutritional Risk Screening [NRS] [26]), disease and multimodal therapy details (i.e., pathological stage [27], details of neoadjuvant and surgical treatments). Postoperative complications were reported according to the Clavien–Dindo severity classification, and severe complications were defined as a Clavien–Dindo degree ≥ 3a. The length of hospital stay was calculated in days between surgery and the date of discharge [28].

OS was defined as the length of time from the operation until death from any cause, and disease-free survival (DFS) was the interval between operation and disease recurrence; disease-specific survival (DSS) referred to the period from the operation until death from GC recurrence or related metastasis, excluding deaths from unrelated causes [29].

Both clinical and therapeutical data were prospectively collected, while details of patient follow-up were periodically updated by a dedicated data manager.

### 2.4. Statistical Analysis

Categorical and continuous variables were presented as numbers (percentages) and means (±standard deviation) unless differently stated. The statistical significance level was set at 0.05, and inference analyses were performed by using the Student’s t-test, Chi-square or Fisher’s exact test, and the Mann–Whitney U test, according to variable types and test assumptions. Long-term outcomes were compared between each group by log-rank test and summarized as Kaplan–Meier curves and hazard ratios with 95% confidence intervals. Statistical analyses were performed by using IBM SPSS Statistics v27.0 (IBM Corp., Armonk, NY, USA).

## 3. Results

### 3.1. Study Population

**Figure 1:**
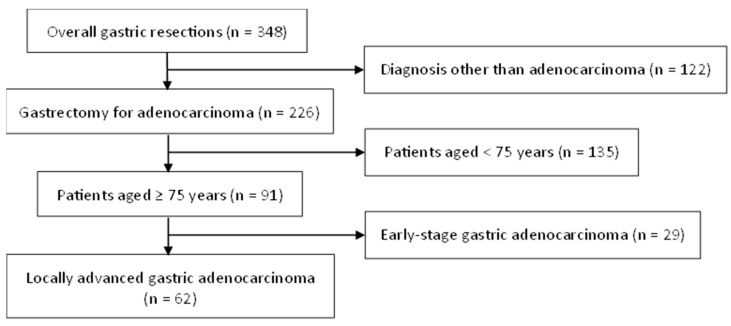
Flowchart of the study group selection.

Overall, 348 consecutive patients were submitted to gastrectomy between January 2015 and December 2020, including 226 patients affected by gastric adenocarcinoma. Among them, 62 presented with an age ≥ 75 years and a clinical stage consistent with LAGC at the time of diagnosis (Figure 1). Gastric resections were total in 29 patients (47%) and distal in 33 (53%), with routine inclusion of D2 lymphadenectomy and omentectomy. According to the patient selection criteria, laparoscopic and open gastrectomy were performed in equal proportions (31 patients each), after a conversion rate of 3.2% (*n* = 1). The open and laparoscopic gastrectomy groups did not significantly differ in terms of demographics, multimodal therapy, histotype, or pathological stage (Table 1).

### 3.2. Primary Outcomes

**Figure 2:**
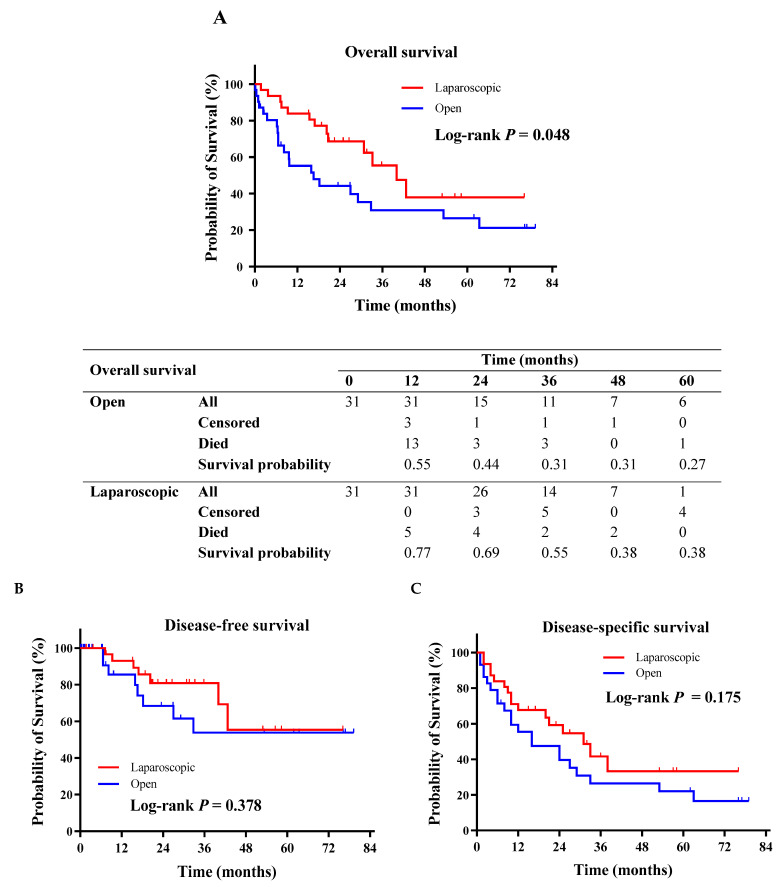
Kaplan–Meier survival analyses. (**A**) Overall survival. (**B**) Disease-free survival. (**C**) Disease-specific survival.

Postoperative complications (Table 2) were significantly lower in the LG group (*p* = 0.039), reporting an overall morbidity as high as 45.2%. Postoperative morbidity occurred in 71% of open GC resections, which also had significantly higher rates of severe (Clavien–Dindo ≥ 3a) pulmonary complications (*p* = 0.038) that required increasing levels of care. The reoperation rate was similar between the open and laparoscopic gastrectomy groups (*p* = 1.000). The LOS after laparoscopic surgery (8 [7,8,9,10] days) was significantly shorter than that after open surgery (12 [9,10,11,12,13,14,15,16,17,18] days), potentially due to the approach-related morbidity burden (*p* = 0.007). After a median follow-up period of 18 months (IQR, 7–34 months), 18 (29%) patients developed disease recurrence and 15 (24%) eventually deceased with evidence of recurrence, with the median time to recurrence being 10 months (IQR, 4–24 months). Overall, 13 (21%) patients deceased for other causes that were not tumor-related. Considering all disease relapses, the patients submitted to laparoscopic gastrectomy showed statistically significant longer 2- and 5-year OS when compared to those submitted to the open approach (0.77 vs. 0.55, and 0.38 vs. 0.27, respectively, *p* = 0.048). Meanwhile, 2- and 5-year DSS did not show differences (0.68 vs. 0.52, and 0.33 vs. 0.20 respectively, *p* = 0.175) according to the surgical approach (Figure 2).

### 3.3. Secondary Outcomes

Intraoperative findings, such as mean operative time and surgical radicality (i.e., marginal tumor involvement and lymph node retrieval), were comparable between the groups. No anesthesia-related adverse events occurred in the entire series. The readmission rate was also similar between the open and laparoscopic gastrectomy groups (*p* = 0.417), with four (12.9%) and two (6.5%) patients requiring further hospitalization, respectively (Table 2).

## 4. Discussion

The present study created the opportunity to assess both the short- and long-term outcomes of a Western series of elderly patients (≥75 years) presenting with LAGC at a national referral center in upper GI cancer surgery. The aim of this analysis was to evaluate the hypothetical effects of the surgical approach on operative stress, which could differently impact surgical outcomes following either laparoscopic or open gastrectomy. As previously postulated in the literature, the findings of this analysis also supported the benefits of a minimally invasive approach to GC surgery in terms of the overall and major (Clavien–Dindo ≥ 3a) postoperative morbidity [30,31,32]. Morbidity after surgery appeared significantly higher following open gastrectomy (*p* = 0.039), and the occurrence of respiratory disorders was significantly different between groups (*p* = 0.038). Pulmonary complications have traditionally been considered one of the most significant achievements of the ERAS protocol application, which has been demonstrated to improve patient recovery by the optimization of clinical determinants affecting the stress response. While providing for an extension of primary tumor resection and lymphadenectomy to the same degree as laparotomy, the minimally invasive approach may entail different interactions on patient functions through an operative stress reduction. Given the standardized clinical and anesthetic management (Supplementary Material Figure S1), the development of pulmonary complications after abdominal surgery can be related to the individual stress response and functional involvement. The type of surgical approach did not affect the main procedural features of gastric resections, and both groups did not report significantly different rates of intraoperative surgical complications accordingly.

The laparoscopic gastrectomy group reported a significantly shorter recovery with a median hospital stay of 8 (7–10) days rather than 12 (9–18) days after open surgery (*p* = 0.007). The fulfillment of standardized discharge criteria depends on a large variety of both clinical and infrastructural factors, from the multispecialty management of the most severe complications to minor events that may increase stress response and recovery. However, accelerated recovery has been widely reported as a postoperative achievement after LG in both the general [33,34] and the elderly population [35,36]. A multicenter study by Honda et al. [37] analyzed an elderly series of patients undergoing any type of laparoscopic gastric resections and reported a minor postoperative morbidity (i.e., pneumonia, intra-abdominal abscess, and anastomotic leakage) with a significantly shorter LOS. A systematic review and meta-analysis by Shan et al. [38] also demonstrated similar benefits among elderly patients, with decreased rates of both respiratory and cardiac complications after LG, with an equivalent anastomotic leakage rate but a significantly shorter LOS when compared to open surgery.

Further evidence has also demonstrated the minimally invasive approach for elderly LAGC patients to provide survival improvements, although the highest rate of compliance with adjuvant treatment could be expected in younger and healthier patients [38,39,40]. Our results demonstrated a longer 5-year OS following laparoscopic gastrectomy (0.38 vs. 0.27, *p* = 0.048) compared to open surgery, which could be considered as the long-term effect of postoperative morbidity on the elderly’s survival, even more than disease recurrence.

The existing literature supports the idea that reducing surgical trauma through a less invasive procedure will preserve the patient’s functional reserve, improving their resistance to severe complications, which is particularly relevant for inherently frail patients.

Studies on the immunological response after LG has shown lower levels of IL-6 and C-reactive protein compared to open surgery, suggesting a reduced impact on the immune system [41,42]. The minimization of inflammation following minimally invasive surgery might contribute to the observed decrease in overall survival due to non-oncologic causes.

Long et al. also reported that LG was linked to an improved 5-year OS for patients aged 60 years and older, with higher rates of postoperative chemotherapy due to a more effective recovery and reduced postoperative morbidity, such as fewer pulmonary complications [43].

To the authors’ interpretation, operative stress reduction and stress response minimization related to the technique’s invasiveness may be crucial to prevent postoperative complications and early mortality in such a delicate class of patients, whose survival could also be limited by several conditions other than tumor disease progression.

The possibility of implementing ERAS pathways in high-risk elderly patients, preserving a good level of compliance with the selected items, has been matter of scientific debate. Previous studies performed in the specific setting of colorectal surgery have also demonstrated the feasibility of adopting fast-track protocols, with an adequate adherence to ERAS items, in elderly patients [44]. However, in oncological gastric surgery, a similar level of scientific evidence is still lacking. In this series, an adequate level of compliance with ERAS protocol was observed in both groups, although laparoscopy has been reported to have an independent role in improving postoperative outcomes, particularly for elderly patients [45]. The implementation of the laparoscopic technique itself was associated with significant modifications in the ERAS pathway (Supplementary Material Table S2), such as a more restrictive fluid balance or less invasive analgesic treatments. Also, the present analysis confirmed the feasibility of LG, for either total or distal resections, in elderly patients. The two groups reported similar proportions of operative time, blood loss, immediate extubation, and admission to the ICU. Despite diffused concerns about octogenarians’ limited tolerance to the increased intrabdominal pressure due to pneumoperitoneum [46,47,48], our series did not report intraoperative adverse events from either respiratory or hemodynamic sides. These findings were consistent with previous analyses proving that CO^2^ pneumoperitoneum did not lead to increased morbidity in elderly GC patients submitted to laparoscopic-assisted distal gastrectomy, even in cases of severe ASA scores [49,50].

The main limitations of the present analysis included the retrospective and single-center study design and the highly selected sample size, especially compared to Eastern studies. In order to minimize the risk of low data accuracy, all cases were consecutively extracted from a prospectively maintained institutional database according to selective inclusion criteria, and a multivariate analysis was also performed. The details of both the perioperative management and the follow-up neither presented significant missing data nor affected the analysis, although both the single-center design and restricted selection criteria limited the number of enrollments and the stratification analyses. However, the choice of a single-center design ensured protocol uniformity of the surgical procedures and perioperative management, which were standardized and equally applied in this study. Eventually, further study limitations may include the following comparative biases: patient selection, the choice of individual surgical approaches, the multidimensional definition of frailty, and specific measurements of stress response variations [38,51].

## 5. Conclusions

Laparoscopic LAGC surgery in elderly cancer patients is feasible and associated with clinical advantages that may impact both short- and long-term outcomes. Based on this analysis, the overall postoperative morbidity was reduced after laparoscopy, with measurable advantages in terms of respiratory complications and LOS. Also, the related procedure-specific means of operative stress minimization showed a favorable impact on elderly patient survival, which is often limited by several causes other than tumor disease or recurrence.

Further multicenter and randomized studies should be designed to explore specific frailty-related areas of weakness/improvement to validate specific risk predictors, and to allocate specific infrastructure resources for elderly GC patients.

## Figures and Tables

**Table 1:** Demographic characteristics of the study population.

Patients	Open 31	Laparoscopic 31	*p*-Value
**Age, median (IQR)**	79 (77–83)	80 (77–82)	0.668
**Gender (Male/Female)**	19/12	18/13	0.796
**ASA, mean (±SD)**	3 (±1)	3 (±1)	0.117
**BMI, mean (±SD)**	25 (±6)	24 (±6)	0.314
**NRS, mean (±SD)**	3 (±1)	3 (±1)	0.102
**aa-CCI, mean (±SD)**	6 (±2)	6 (±2)	0.940
COPD	2 (6.5%)	2 (6.5%)	1.000
CHF	2 (6.5%)	3 (9.7%)	0.640
Blood hypertension	15 (48.4%)	9 (29%)	0.118
Diabetes	4 (12.9%)	3 (9.7%)	0.688
Preoperative hemoglobin, mean (SD)	11.2 (±2.4)	11.7 (±2.6)	0.260
Preoperative serum creatinine, mean (SD)	1.1 (±0.4)	1 (±0.3)	0.460
**Histotype**			
SRCC	6 (19.4%)	9 (29%)	0.374
non-SRCC	25 (80.6%)	22 (71%)	
**Neoadjuvant treatment**			
Chemotherapy	4 (12.9%)	4 (12.9%)	1.000
Regimen:			
- FLOT	1/4 (25%)	1/4 (25%)	0.503
- ECF	2/4 (50%)	1/4 (25%)	
- TCF	1/4 (25%)	0	
- FOLFOX	0	2/4 (50%)	
- Other			
Completion rate	3/4 (75%)	3/4 (75%)	1.000
**Surgery**			
Total gastrectomy	13 (41.9%)	16 (51.6%)	0.268
Distal gastrectomy	18 (58.1%)	15 (48.4%)	0.346
Conversion to open		1 (3.2%)	
**TNM stage**			
LAGC	31 (100%)	31 (100%)	1.000
N > 0	24 (77.4%)	19 (61.3%)	0.168
T > 3	11 (35.5%)	10 (32.3%)	0.788

IQR, interquartile range; SD, standard deviation; ASA, American Society of Anesthesiologists’ classification of physical health; BMI, body mass index; NRS, nutritional risk screening score; aa-CCI, age-adjusted Charlson comorbidity index; COPD, chronic obstructive pulmonary disease; CHF, congestive heart failure; SRCC, signet-ring cell adenocarcinoma; ECF, epirubicin, cisplatin, and 5-fluorouracil; TCF, docetaxel, cisplatin, and 5-fluorouracil; FLOT, fluorouracil, leucovorin, oxaliplatin, and docetaxel; FOLFOX, folinic acid, fluorouracil, and oxaliplatin; LAGC, locally advanced gastric cancer based on clinical TNM staging system.

**Table 2:** Perioperative outcomes.

Patients	Open 31	Laparoscopic 31	*p*-Value
**Intraoperative data**			
Operative time (minutes), mean (SD)	190 (±65)	216 (±60)	0.058
Lymph node retrieval, median (IQR)	37 (25–45)	37 (28–53)	0.660
**Postoperative data**			
Complications:			
- Overall	22 (71%)	14 (45.2%)	0.039
- Severe (Clavien–Dindo ≥ 3a)	9 (29%)	5 (16.1%)	0.224
- Anastomotic leak	3 (9.7%)	3 (9.7%)	1.000
- Duodenal stump leak	3 (9.7%)	1 (3.2%)	0.291
- Respiratory	3 (9.7%)	0	0.038
- Cardiac	0	0	-
- Urinary	1 (3.2%)	0	0.236
- Wound infection	2 (6.5%)	0	0.092
Reoperation	2 (6.5%)	2 (6.5%)	1.000
Indication to reoperation:			
- Anastomotic leak	1 (3.2%)	1 (3.2%)	1.000
- Duodenal stump leak	1 (3.2%)	1 (3.2%)	1.000
30-day mortality	1 (3.2%)	1 (3.2%)	1.000
30-day readmission	4 (12.9%)	2 (6.5%)	0.417
Length of hospital stay, median (IQR)	12 (9–18)	8 (7–10)	0.007

SD, standard deviation; IQR, interquartile range.

## Data Availability

Data are available upon request.

## Supplementary Materials

The following supporting information can be downloaded at: https://www.mdpi.com/article/10.3390/cancers16132477/s1, Figure S1: ERAS-based perioperative protocol for gastrectomy; Table S1: STROBE Statement—Checklist of items that should be included in reports of cohort studies; Table S2: Anesthesiology management according to surgical approach.

## Appendix A

The OSR CCeR Collaborative Group: Barbieri Lavinia A. (Department of Gastrointestinal Surgery, IRCCS San Raffaele Scientific Institute, 20132 Milan, Italy), Cossu Andrea (Department of Gastrointestinal Surgery, IRCCS San Raffaele Scientific Institute, 20132 Milan, Italy), Treppiedi Elio (Department of Gastrointestinal Surgery, IRCCS San Raffaele Scientific Institute, 20132 Milan, Italy), Battaglia Silvia (Department of Gastrointestinal Surgery, IRCCS San Raffaele Scientific Institute, 20132 Milan, Italy), Cusin Sofia (Department of Gastrointestinal Surgery, IRCCS San Raffaele Scientific Institute, 20132 Milan, Italy).
