# Supplementary material for: Short- and Long-Term Advantages of Laparoscopic Gastrectomy for Elderly Patients with Locally Advanced Cancer
Source: Cancers (Basel). 2024 Jul 7;16(13):2477. doi: 10.3390/cancers16132477 (PMC11240721; doi:10.3390/cancers16132477)
Supplement: Supplementary file 1 [file cancers-16-02477-s001.zip › Table S2.pdf]

Table S2. Anesthesiology management according to surgical approach

Intraoperative ERAS items	Laparoscopic gastrectomy	Laparotomic gastrectomy
**Pain management**	Multimodal minimally invasive protocol:<br>• Wound infiltration with local anaesthetics<br>• PCA of morphine with injection dose 1mg, lockout time 10min, maximum dosage 4mg/h.<br>• Multimodal analgesic adjuvant therapy	Thoracic epidural analgesia:<br>• T7-T9<br>• 0.2% Ropivacaine + Sufentanil 0.5mcg/mL (no bolus, only continuous infusions)<br>• 4-6 ml/h up to POD 3-4
**Goal-directed fluid therapy**	Target: SVV < 10% with MAP > 70mmHg<br>Basal infusion rate: 4-5ml/kg/h	Target: SVV < 10% with MAP > 70mmHg<br>Basal infusion rate: 7-8ml/kg/h
