# Supplementary figures and images for: Short- and Long-Term Advantages of Laparoscopic Gastrectomy for Elderly Patients with Locally Advanced Cancer
Source: Cancers (Basel). 2024 Jul 7;16(13):2477. doi: 10.3390/cancers16132477 (PMC11240721; doi:10.3390/cancers16132477)

**Figure S1. ERAS-based perioperative protocol for gastrectomy**

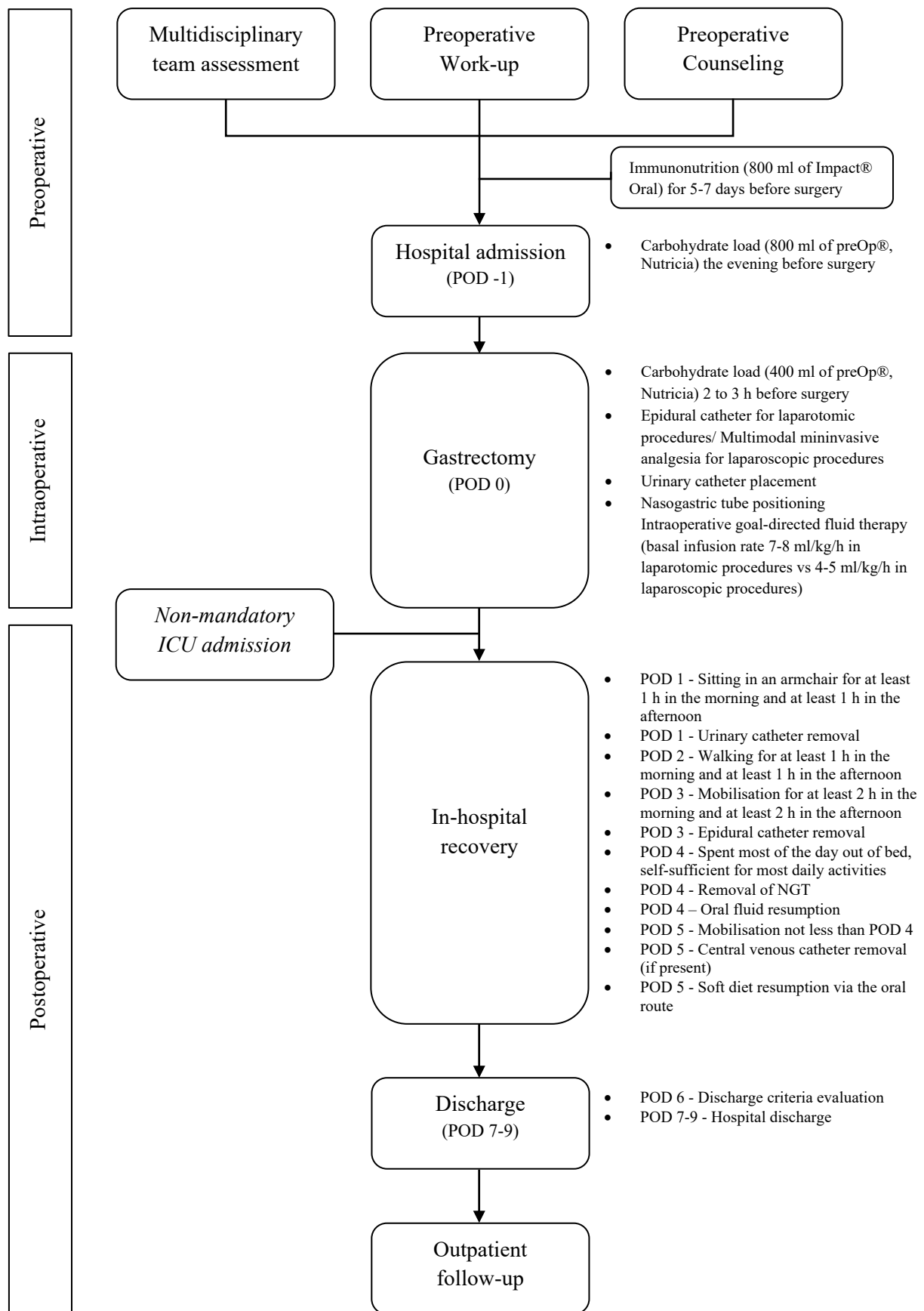

Supplement: Supplementary file 1 [file cancers-16-02477-s001.zip › Figure S1.pdf]
